# Host’s demand for essential amino acids is compensated by an extracellular bacterial symbiont in a hemipteran insect model

**DOI:** 10.3389/fphys.2022.1028409

**Published:** 2022-09-30

**Authors:** Minoru Moriyama, Takema Fukatsu

**Affiliations:** ^1^ Bioproduction Research Institute, National Institute of Advanced Industrial Science and Technology (AIST), Tsukuba, Japan; ^2^ Department of Biological Sciences, Graduate School of Science, University of Tokyo, Tokyo, Japan; ^3^ Graduate School of Life and Environmental Sciences, University of Tsukuba, Tsukuba, Japan

**Keywords:** essential amino acids, extracellular symbiosis, gut bacterial symbiont, nutritional mutualism, phloem sap

## Abstract

Plant sap is a nutritionally unbalanced diet that constitutes a challenge for insects that feed exclusively on it. Sap-sucking hemipteran insects generally overcome this challenge by harboring beneficial microorganisms in their specialized symbiotic organ, either intracellularly or extracellularly. Genomic information of these bacterial symbionts suggests that their primary role is to supply essential amino acids, but empirical evidence has been virtually limited to the intracellular symbiosis between aphids and *Buchnera*. Here we investigated the amino acid complementation by the extracellular symbiotic bacterium *Ishikawaella* harbored in the midgut symbiotic organ of the stinkbug *Megacopta punctatissima*. We evaluated amino acid compositions of the phloem sap of plants on which the insect feeds, as well as those of its hemolymph, whole body hydrolysate, and excreta. The results highlighted that the essential amino acids in the diet are apparently insufficient for the stinkbug development. Experimental symbiont removal caused severe shortfalls of some essential amino acids, including branched-chain and aromatic amino acids. *In vitro* culturing of the isolated symbiotic organ demonstrated that hemolymph-circulating metabolites, glutamine and trehalose, efficiently fuel the production of essential amino acids. Branched-chain amino acids and aromatic amino acids are the ones preferentially synthesized despite the symbiont’s synthetic capability of all essential amino acids. These results indicate that the symbiont-mediated amino acid compensation is quantitatively optimized in the stinkbug-*Ishikawaella* gut symbiotic association as in the aphid-*Buchnera* intracellular symbiotic association. The convergence of symbiont functions across distinct nutritional symbiotic systems provides insight into how host-symbiont interactions have been shaped over evolutionary time.

## Introduction

Obligatory dependence on endosymbiotic microorganisms has evolved repeatedly and independently in a wide variety of insects (Baumann, 2005; Bennett and Moran et al., 2015; Douglas, 2015; Salem and Kaltenpoth, 2022). Versatile abilities of microbial symbionts, such as nutritional provisioning (Douglas, 2009; Hansen et al., 2020), degradation of indigestible materials (Brune, 2014; Salem et al., 2020), and synthesis of protective chemicals (Flórez et al., 2015); enable insects to adapt to a wider range of environments, thereby facilitating their propagation and diversification. The host insects often develop specialized organs to harbor particular symbionts intracellularly or extracellularly, and to ensure the transmission of the microbial assets to their offspring (Buchner, 1965; Baumann, 2005; Engel and Moran, 2013; Salem et al., 2015; Hosokawa and Fukatsu, 2020).

To date, many genomes of symbiotic bacteria have been diligently sequenced. It revealed symbiont genome reduction along with extensive gene loss emerges as a general rule while some meaningful gene sets persist, thereby enabling inference for presumable symbiont functions (Moran et al., 2008; McCutcheon and Moran, 2012). Among diverse symbionts whose host insects feed exclusively on plant sap (Douglas, 2006; Dinant et al., 2010), the reduced genomes retain the genes for synthesis of essential amino acids (EAAs) and vitamins in general, which implies the pivotal role of these symbionts in providing essential nutrients that the host insects are unable to synthesize (Shigenobu et al., 2000; McCutcheon et al., 2009; Nikoh et al., 2011; Kaiwa et al., 2014; Chong et al., 2019). It is also often observed that even within a single metabolic pathway, several genes that can be replaced by host genes have been lost in the symbiont genome, suggesting nutrient production is attained by cooperative host-symbiont metabolic interactions (Wilson et al., 2010; Hansen and Moran, 2011; Russel et al., 2013).

While genomic data have provided valuable evolutionary insights into the host-symbiont relationships, functional inference based merely on gene repertoire suffers fundamental limitation, especially in quantitative aspects. Even if a symbiont possesses a genome-coded metabolic capability for synthesizing all EAAs, yields of each EAA should be fine-tuned depending on the feeding ecology of its host insect, because the nutrient composition of plant sap may vary depending on plant species, plant parts, and other environmental conditions (Sandström and Pettersson, 1994; Sandström and Moran, 2001; Dinant et al., 2010; Selvaraj et al., 2021). In addition, recently acquired or replaced symbionts of relatively young evolutionary origin tend to have less reduced genomes, where the host-symbiont gene redundancies have not been fully streamlined, but such symbionts still offer clear fitness advantages to their host insects (Oakeson et al., 2014; Hosokawa et al., 2016; Husnik and McCutcheon, 2016; Chong and Moran, 2018; Otero-Bravo and Sabree, 2021). For these systems, it is difficult to narrow down the plausible symbiont roles merely based on their gene repertoire. Therefore, experimental validation and quantitative evaluation of nutritional interactions are very important and to be performed in parallel with the genomic approaches.

Nevertheless, such integrative studies have been practically restricted to a single model system, aphids and their intracellular symbiotic bacteria *Buchnera* (Douglas, 1998; Wilson, 2020). *Buchnera* is housed in specialized aphid cells called the bacteriocytes, and both nutritional and genomic studies have verified that its primary role is provisioning of EAAs and vitamins, which enables aphid propagation on a nutritionally imbalanced diet of plant phloem sap (Douglas, 2006). The levels of individual EAA production are considered to be regulated by the precursor flow from the host cell to the symbiont cells, rather than by transcriptional regulation of the symbiont genes (Moran et al., 2005; Reymond et al., 2006; MacDonald et al., 2011; Smith and Moran., 2020). Hence, it is of pivotal importance to understand the metabolic interactions across the host-symbiont interface. In this context, it is also of great interest how such host-symbiont metabolic interactions are operating in extracellular symbiotic systems, in which the symbiont cells are housed in extracellular spaces constituted by the host cell layer, where the host-symbiont interface and nutrient exchange entail distinct structural and cellular configurations.

**FIGURE 1:**
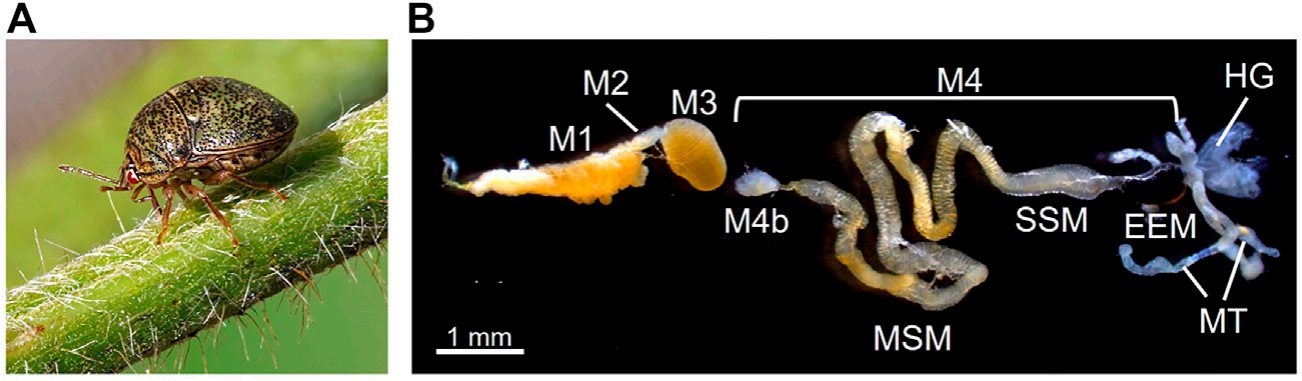
Extracellular gut symbiotic system of the stinkbug *M. punctatissima*. **(A)** An adult insect feeding on kudzu plant. **(B)** The alimentary tract dissected from an adult female. The first three midgut sections (M1-M3) are involved in food digestion and absorption. The uncultivable bacterial symbiont *Ishikawaella* is housed in the fourth midgut section (M4), which is further divided into the M4 bulb (M4b), main symbiotic midgut (MSM), swollen symbiotic midgut (SSM), and enlarged end midgut (EEM). The posterior end of M4 is connected to the Malpighian tubeles (MT) and hindgut (HG). Males lack MSM and EEM for production of symbiont-containing capsules for vertical transmission (see Hosokawa et al., 2005; Koga et al., 2021).

In this context, we aim at experimental validation of EAA compensation using an extracellular bacterial symbiont of a plataspid stinkbug as a model system. The kudzu bug *Megacopta punctatissima* (species complex with *M. cribraria*; see Hosokawa et al., 2014) (Figure 1A) propagates primarily on kudzu plants (*Pueraria montana* var. *lobata*) but can utilize many other leguminous plants, including soybean (*Glycine max*) crops (Eger et al., 2010; Blount et al., 2015). Recent invasions and rapid spread of this species into the North American continent threaten the agricultural economy of soybean fields (Ruberson et al., 2012; Dhammi et al., 2016). The stinkbug primarily utilizes phloem fluid (Stubbins et al., 2017), and is likely to suffer imbalanced nutrition, such as excess sugar and shortage of EAAs, as in aphids (Douglas, 2006; Dinant et al., 2010). *M. punctatissima* is obligatorily associated with a gut bacterial symbiont “*Candidatus* Ishikawaella capsulata” (hereafter called *Ishikawaella*), which is indispensable for normal growth, reproduction, behavior, and plant utilization of the host insect (Fukatsu and Hosokawa, 2002; Hosokawa et al., 2006, 2007, 2008; Brown et al., 2014; Koga et al., 2021). The posterior portion of the midgut is highly developed and differentiated for housing a large symbiont population in its lumen (Figure 1B) (Fukatsu and Hosokawa, 2002; Hosokawa et al., 2005, 2006; Koga et al., 2021). This symbiotic midgut is structurally separated from the anterior digestive midgut and thus, oddly, specialized for symbiont retention without food flow (Ohbayashi et al., 2015; Oishi et al., 2019). The mother stinkbugs transmit the symbiotic bacteria to the next generation by producing symbiont-containing capsule-like particles together with the eggs, and newly hatched nymphs obtain the symbiont by oral ingestion of the capsule content (Fukatsu and Hosokawa, 2002; Hosokawa et al., 2005, 2007; Koga et al., 2021). Experimental removal of the symbiotic bacteria causes severe growth retardation and abnormal morphology of the symbiont-free insects (Fukatsu and Hosokawa, 2002; Hosokawa et al., 2006; Koga et al., 2021). The *Ishikaella* genome sequencing revealed reduced size (0.75 Mb), loss of many metabolic genes, and retention of genes involved in all EAA synthesis pathways (Nikoh et al., 2011). Based on the genomic information, therefore, *Ishikawaella* is responsible for provisioning of EAAs, which is similar to the role of *Buchnera* in aphids, despite their distinct symbiotic configurations.

In this study, we performed quantitative experimental studies on the amino acid metabolism in the *M. punctatissima*-*Ishikawaella* gut symbiotic system. In particular, we evaluated the nutritional adequacy of the diet, the effect of symbiont removal on nutritional balance, and the differential production of EAAs in the symbiotic organ under *in vitro* conditions. The results verified the biased EAA production as a result of cooperative host-symbiont metabolic interactions, which provided the basic framework to establish an extracellular model system for investigating nutritional symbiosis.

## Materials and methods

### Insects

Adults and nymphs of *M. punctatissima* on field kudzu plants were collected in Tsukuba, Ibaraki, Japan. Some adult insects were maintained on young pea pods (*Pisum sativum*) at 25 °C under a long day regime of 16 h light and 8 h dark and allowed to lay eggs (Hosokawa et al., 2007). Hatched nymphs were reared on soybean seedlings up to getting the adult stage. To obtain symbiont-free insects, symbiont-containing capsules were removed from egg masses by fine forceps (Hosokawa et al., 2006), and resultant symbiont-free hatchlings were reared on soybean seedlings as described above.

### Collection of hemolymph, excreta, and plant sap

Hemolymph was collected from the neck of adults by piercing the intersegmental membrane. Bleeding hemolymph was quantitatively retrieved using a glass capillary (Microcaps 1 μL, Drummond), merged with 50 μL of IS solution (water containing 400 pmol/μL of norvaline and sarcosine as internal standards), and stored at -80°C until analysis. For collection of excreta, adults were transferred to a new plastic Petri dish (9 cm diameter, 1.5 cm depth) whose bottom was covered with a water-repelling plastic film (Parafilm). For nymphs, a small piece of parafilm was tied on the stem underneath the abdominal tip of each of fourth instar nymphs to receive their droppings. We carefully monitored them, and 5–10 μL of freshly dropped excreta were collected with a micropipette and processed as described for hemolymph.

Plant phloem sap was collected using the ethylenediaminetetraacetic acid (EDTA)-facilitated exudation method (Neo and Layzell, 1997). A petiole of kudzu or soybean plant was cut, immersed in 1 ml of 5 mM EDTA solution (pH 6.0), and preincubated for 60 min under a humid condition. Then, the petiole was immersed in 200 μL of new EDTA solution, covered with misted plastic wrap, and kept for 4 h under darkness. The exudate samples were lyophilized and stored at -80°C.

### Free and protein amino acids in nymphal bodies

Symbiotic and aposymbiotic nymphs reared on soybean seedlings were collected at the fourth instar stage, and their fresh weight was recorded. Some nymphs were homogenized in 100 μL of the IS solution, filtrated by a 0.22 μm filter (Advantec), and subjected to the amino acid analysis as described below. To hydrolyze protein amino acids, some nymphs were homogenized in 100 μL of 6 N hydrochloric acid solution containing 0.1% (w/v) phenol, and heated at 110 °C for 24 h. After neutralization by an addition of 4 N sodium hydroxide solution, the hydrolysate was passed through a 0.22 μm filter. Its 1/50 aliquot was merged with 50 μL of IS solution and subjected to amino acid analysis as described below.

### *In Vitro* culture of symbiotic midgut

We used a culture medium modified from Buffer A (Sasaki and Ishikawa, 1995). The base composition of the medium was 25 mM KCl, 10 mM MgCl_2_, and 35 mM 4-(2-hydroxyethyl)-1-piperazineethanesulfonic acid (HEPES, pH 7.4). The base medium was supplemented with 200 mM sugar (cellobiose, sucrose, trehalose, or mannitol) and/or 0.1–10 mM amino acid (glutamine or glutamate). In tracer experiments, [2–^15^N]-labeled glutamine (Cambridge Isotope Laboratories) was used. All components were sterilized through a 0.22 μm filter. The symbiotic midgut preparations were dissected from adult females of *M. punctatissima*. About 1 mm segment of the symbiotic midgut was ligated with fine polyethylene thread (*ϕ* 300 μm) at both ends and cut at the outer side of the knots. Each of the ligated preparations was transferred to a medium drop hanging on a 24-well (ϕ 4 mm) glass slide (Matsunami Glass Ind.). To remove carry-over effects of internal amino acids, the preparations were first preincubated in the medium containing no amino acids for 30 min at 25°C. Then, the medium was replaced with 15 μL of a new test medium, and incubated for 2 h at 25°C. Then the medium was collected and stored at -80°C until analysis.

### Amino acid quantification

Amino acid compositions were analyzed by high-performance liquid chromatography (HPLC) or liquid chromatography and mass spectrometry (LC-MS). For HPLC quantification, we used Agilent 1100 system equipping a diode array detector. Amino acids were derivatized using o-phthalaldehyde and 9-fluorenylmethyl chloroformate regents according to a supplier’s protocol (Agilent application 5980-1193). The derivatized amino acids were separated on Zorbax Eclipse-AAA column (Agilent, 75 mm × 4.6 mm i. d.) at a flow rate of 2 ml/min. Solvent A was 40 mM phosphate buffer at pH 7.8 while solvent B was acetonitrile/methanol/water = 45/45/10 (v/v/v). The proportion of solvent B increased from 0 to 57% in 10 min. The absorption levels were monitored at 338 and 262 nm.

For the culture samples, we used an LC-MS system consisting of the Shimadzu Prominence series and Thermo LCQ Duo with an electrospray ionization (ESI) source. Released amino acids in the culture medium were subjected to derivatization with propyl chloroformate (Uutela et al., 2009). The culture medium was mixed with 50 μL of 0.2 M borate buffer (pH 10.2) containing 50–500 pmol internal standards (homoarginine and homophenylalanine), and then with 40 μL of pyridine:1-propanol (1:4) solution. Alkylation was yielded by adding 25 μL of 20% (v/v) propylchroloformate in chloroform. Derivatized amino acids were extracted with 100 μL chloroform, dried, and resuspended to 50% (v/v) methanol. The samples were separated on a Shim-pack FC-ODS column (150 mm × 2 mm i. d., Shimadzu) at a flow rate of 0.2 ml/min using a gradient elution protocol; Mobile phase A was water containing 0.05% formic acid and 2.5 mM ammonium formate, while mobile phase B was methanol, and the concentration of mobile phase B was increased from 56 to 90% in 13 min. Protonated ions [M + H]^+^ of each amino acid were monitored in a positive ESI mode. The quantity of ^15^N-labeled amino acids was calculated by subtracting the naturally occurring isotope ratio.

## Results and discussion

### Amino acid sufficiency in diets

**FIGURE 2:**
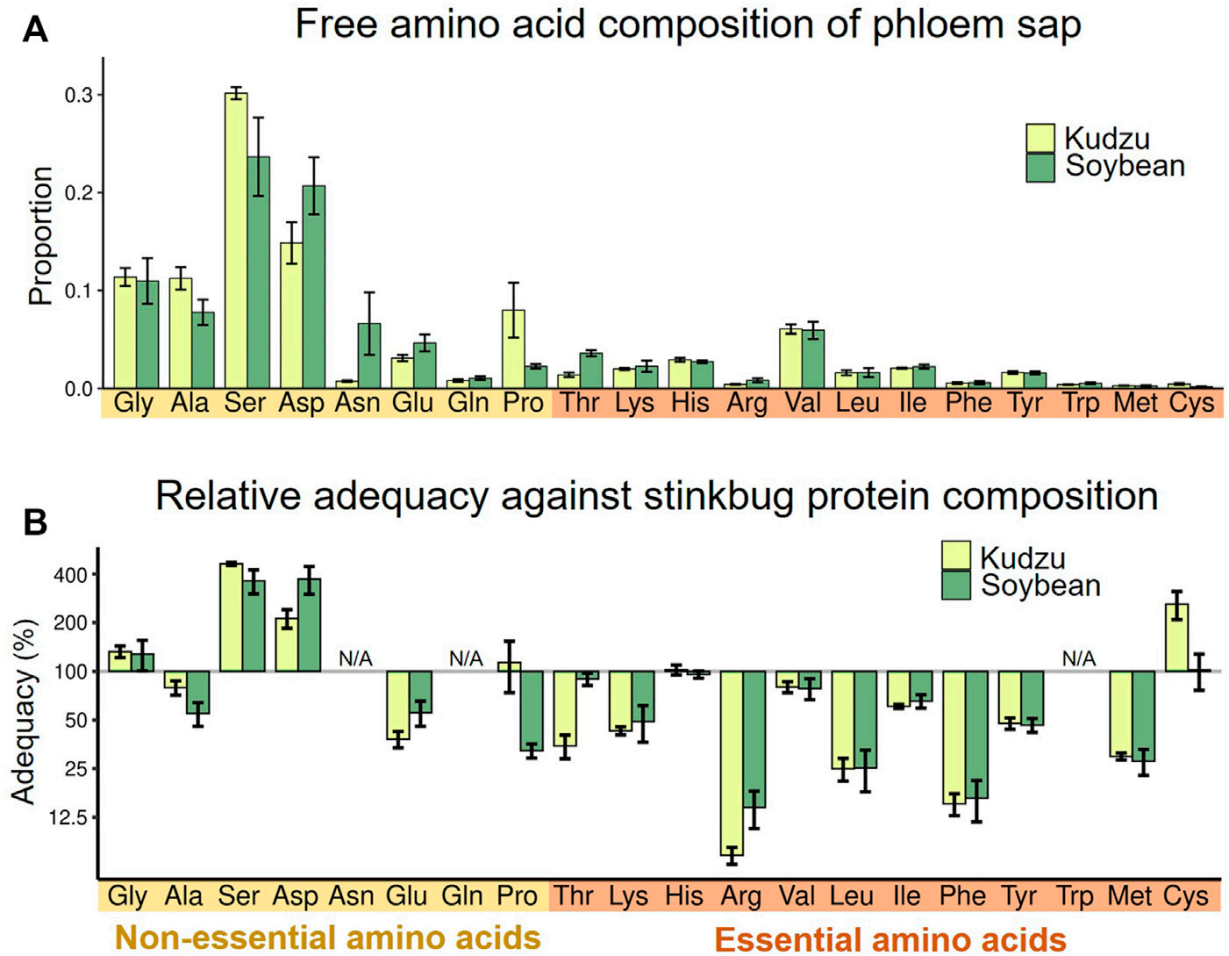
Nutritional evaluation of dietary plant sap. **(A)** Relative proportion of amino acids in the phloem sap of two host plants, kudzu and soybean. Means ± s.e.m are shown (*N* = 6). **(B)** Amino acid adequacy of the phloem saps for stinkbug development. The relative adequacy was calculated against the protein amino acid composition of the whole body of fourth instar nymphs (see also Supplementary Table S1). The amounts of asparagine and glutamine were incorporated into those of aspartate and glutamate, respectively, while tryptophan was omitted, because they were processed during protein hydrolysis (N/A). Means ± s.e.m are shown at the logarithmic scale (*N* = 6).

First, we evaluated the amino acid compositions of plant phloem sap as nutritional sources for *M. punctatissima*. Samples of plant sap were extracted from wild kudzu plants and soybean seedlings using an EDTA-facilitated exudation method (Neo and Layzell, 1997). The overall amino acid compositions detected in the kudzu and soybean sap samples were similar to each other (Figure 2A). Serine and aspartate were the two major amino acids. Proline was higher in kudzu than in soybean, while asparagine was the opposite. In both plant sap samples, the proportions of non-EAAs were 78–80%, which outweighed those of EAAs. Because EAAs comprised 44.3% of the body proteins of *M. punctatissima* (see Supplementary Table S1), this strong bias toward non-EAAs in the plant sap diet suggests that the stinkbug faces a persistent EAA shortage, as documented in other phloem sap-feeding insects (Douglas, 2006). When we calculated the relative adequacy of individual amino acids against the body protein composition, most EAAs, especially arginine, leucine, and phenylalanine, were predicted to be insufficient to support stinkbug growth (Figure 2B; Supplementary Table S1). It should be noted that, in this paper, we regarded two semi-essential amino acids, tyrosine and cysteine, as EAAs, because they must be derived from other EAAs, phenylalanine and methionine, respectively. These amino acids are highly restricted in the plant sap.

**FIGURE 3:**
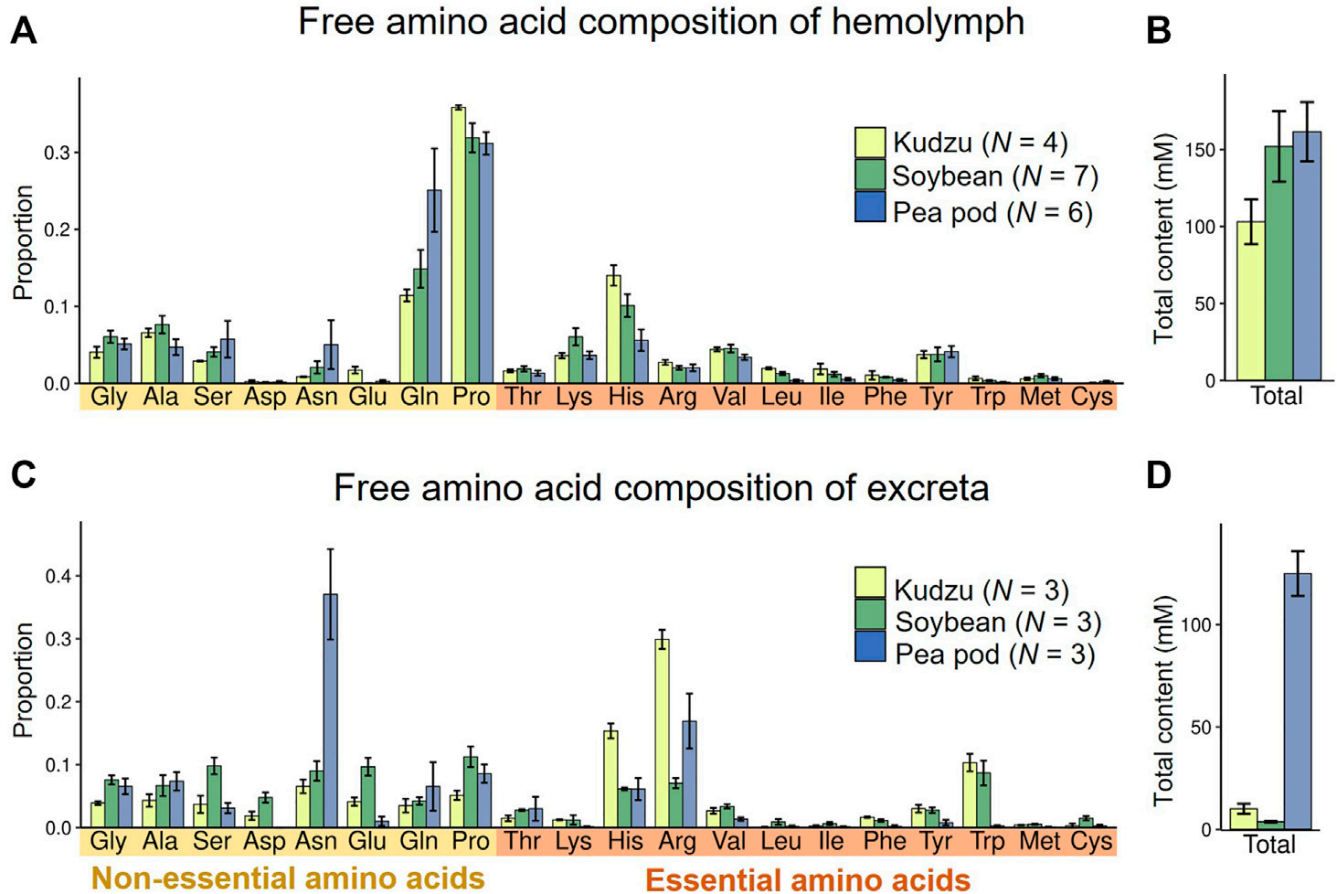
Amino acid compositions in hemolymph and excreta of *M. punctatissima* adults feeding on different diets, kudzu plant, soybean seedling, and pea pod. **(A,B)** Relative proportion of each amino acid **(A)** and total amount of amino acids **(B)** in the hemolymph. Means ± s.e.m are shown (*N* = 4-7). **(C,D)** Relative proportion of each amino acid **(C)** and total amount of amino acids **(D)** in excreta. Means ± s.e.m are shown (*N* = 3).

Next, we investigated amino acid compositions of hemolymph of *M. punctatissima* adults reared on different food plants, namely kudzu plants, soybean seedlings, and pea pods. The overall amino acid compositions and quantities were similar irrespective of the food plants (Figures 3A,B), but they were conspicuously deviated from the amino acid compositions of the phloem sap samples (Figure 2A). Proline and glutamine were the most abundant amino acids in the hemolymph. In contrast, serine and aspartate, which were rich in phloem sap, were scarce (Figure 3A). The hemolymph of *M. punctatissima* contained trehalose as a dominant sugar, but not sucrose (Supplementary Table S2), as in many other insects (Moriwaki and Matsushita, 2003; Thompson, 2003). These patterns indicate that the hemolymphal nutritional state is under physiological and metabolic control to serve for nutrient exchange across a variety of body organs, rather than simply reflecting the dietary state.

In contrast to the homeostatic patterns in the hemolymphal amino acids, the amino acid compositions and quantities of excreta significantly varied with food sources (Figures 3C,D). Outstandingly high levels of amino acids were detected in the excreta of pea pod-sucking insects (Figure 3D), and asparagine accounted for one-third of them (Figure 3C). Although the amino acid composition of pea pod sap was not measured in this study (see Figure 2), it is reported that a high concentration of amino acids is circulating in young legume pods to deliver nutrients for seed development (Atkins et al., 1975; Rochat and Boutin, 1991; Ohyama et al., 2017). Feeding on bean pods is observed but unusual in *M. punctatissima* in the field (Ruberson et al., 2012). Therefore, it seems likely, although speculative, that the forced feeding on pea pods might have caused an overload of amino acids and resulted in the excess discharge amino acids in the excreta. Irrespective of the food sources, arginine and histidine represented the preferentially excreted EAAs (Figure 3C). In contrast, three branched-chain amino acids (valine, leucine, isoleucine), two aromatic amino acids (phenylalanine, tyrosine), lysine, and methionine were scarcely discharged (Figure 3C), which suggest that these amino acids may be insufficient and required for *M. punctatissima*.

### Symbiont-mediated nutritional compensation

**FIGURE 4:**
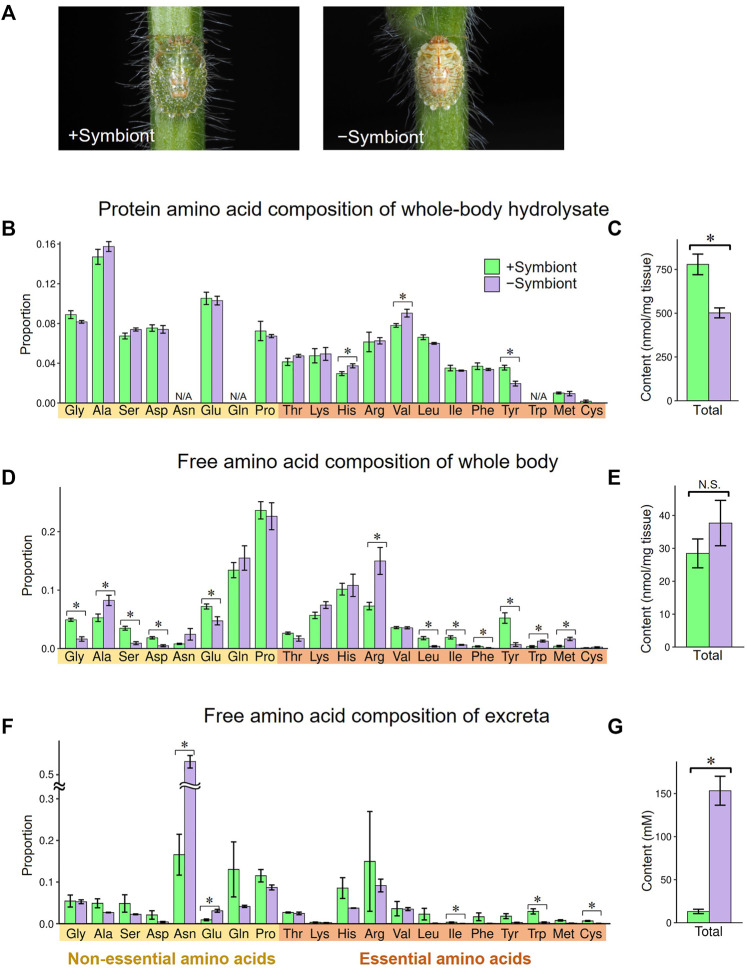
Effects of symbiont removal on amino acid profiles in fourth instar nymphs of *M. punctatissima*. **(A)** External appearance of symbiotic (left) and aposymbiotic (right) fourth instar nymphs. **(B,C)** Protein amino acids from the whole body. **(D,E)** Free amino acids from the whole body. **(F,G)** Free amino acids in the excreta. **(B,D,F)** Relative proportion of each amino acid. **(C,E,G)** Total amount of amino acids. For **(B)**, the amounts of asparagine and glutamine were incorporated into aspartate and glutamate, respectively, while that of tryptophan was omitted (N/A) due to the hydrolysis treatment. Means ± s.e.m are shown (*N* = 6 for **(B–E)**, *N* = 3 for **(F,G)**). Asterisks indicate statistically significant differences (*p* < 0.05, *t*-test).

To evaluate the influence of the gut symbiont *Ishikawaella* on the nutritional physiology of the host *M. punctatissima*, we compared amino acid flows between normal *Ishikawaella*-infected (= symbiotic) insects and uninfected (= aposymbiotic) insects. The aposymbiotic insects were generated by removing symbiont-containing capsules from egg masses (Hosokawa et al., 2006). Both the symbiotic and aposymbiotic nymphs were reared on soybean seedlings, and fourth instar nymphs were subjected to the analyses on account of the low adult emergence rate of aposymbiotic insects (Hosokawa et al., 2006). The aposymbiotic nymphs showed paler color, slower growth, lower survival, and smaller body size in comparison with the symbiotic nymphs (Figure 4A) (Fukatsu and Hosokawa, 2002; Hosokawa et al., 2006). When protein amino acid compositions of the whole body were compared, the aposymbiotic nymphs exhibited less proteinous constituents per wet weight than the symbiotic nymphs, whereas overall amino acid compositions were similar between the aposymbiotic and symbiotic nymphs (Figures 4B,C). A notable exception was tyrosine, whose amount in the aposymbiotic nymphs was about half in comparison with the symbiotic nymphs (Figure 4B). More drastic effects were seen in the free amino acid compositions of the whole body. While no significant difference was detected in the total amino acid quantities, two branched-chain amino acids (leucine and isoleucine) and two aromatic amino acids (phenylalanine and tyrosine) significantly decreased in the aposymbiotic nymphs (Figures 4D,E). In contrast, the aposymbiotic nymphs exhibited higher titers for several EAAs such as arginine, tryptophan, and methionine (Figure 4D).

Notably, the aposymbiotic nymphs excreted an overwhelmingly large amount of amino acids, which was mainly accounted for by asparagine, in comparison with the symbiotic nymphs (Figures 4F,G). The relative concentrations of most EAAs, including branched-chain amino acids and aromatic amino acids, were lower in the aposymbiotic nymphs than in the symbiotic nymphs, although the differences were not always significant statistically probably due to small sample sizes (Figure 4F). Similarly, previous studies reported that aposymbiotic aphids excrete higher levels of glutamine and asparagine than normal aphids (Sasaki et al., 1990; Prosser and Douglas, 1991). It can be explained by the process that the disruption of symbiont-mediated conversion from non-EAAs to EAAs ends up with accumulation and excretion of non-EAA. As shown in Figure 3C, *M. punctatissima* selectively excreted a large amount of asparagine presumably as nitrogenous waste (Figure 4F), although its hemolymphal titer was low (Figure 4D). Asparagine seems to be a suitable non-EAA for nitrogenous waste disposal because it contains two nitrogen atoms per molecule. All these results taken together, it is suggested that *Ishikawaella* compensates for the shortage of dietary EAAs, especially branched-chain amino acids and aromatic amino acids, by which the host stinkbug can efficiently utilize the plant sap nutrients.

### Amino acid production in midgut symbiotic organ

**FIGURE 5:**
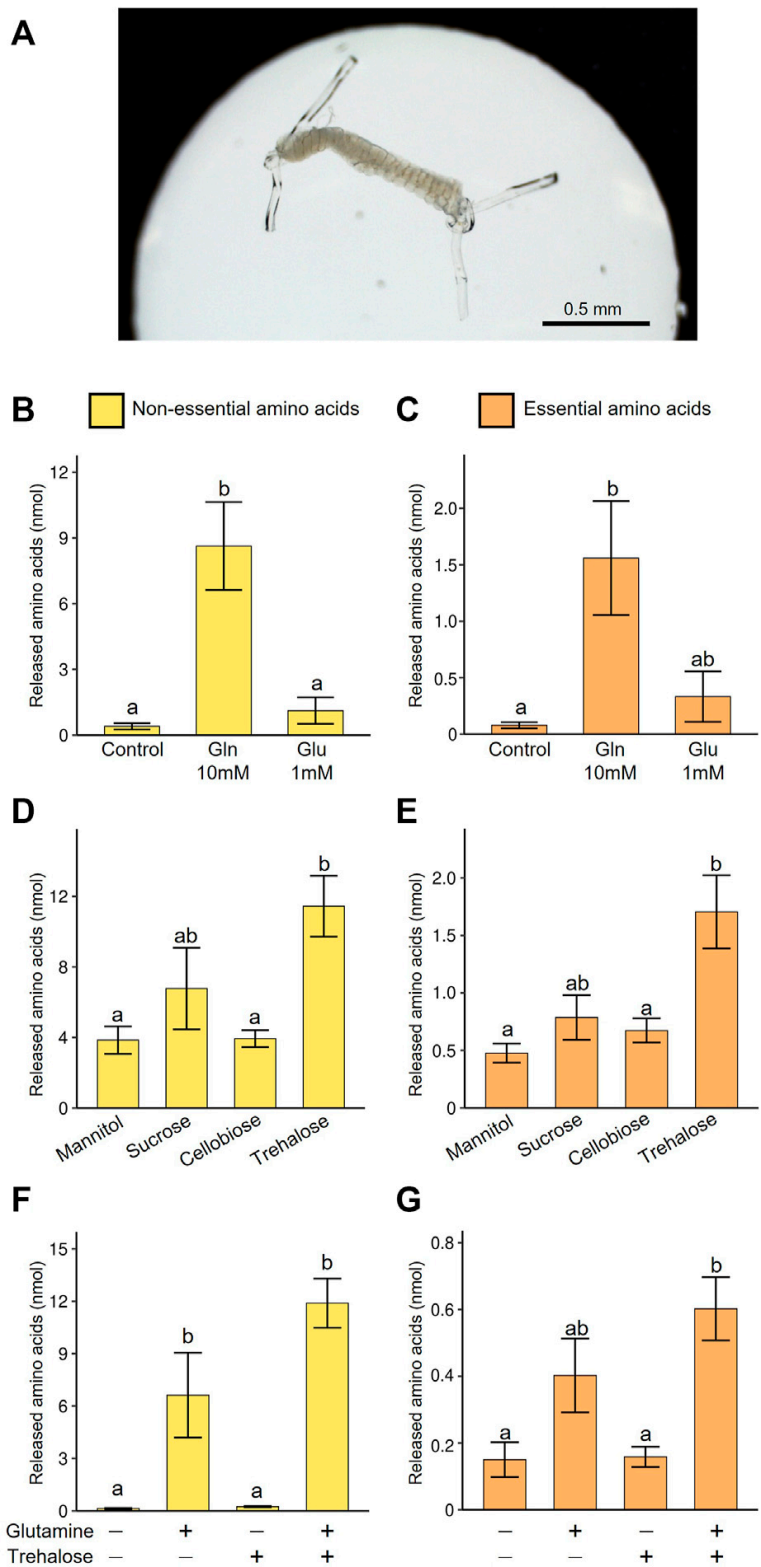
Amino acid production by the isolated midgut symbiotic organ of *M. punctatissima*. **(A)** An isolated symbiotic midgut in a medium drop. **(B,C)** Effect of nitrogen sources (10 mM glutamine or 1 mM glutamate) on production of non-essential amino acids **(B)** and essential amino acids **(C)** in the presence of 200 mM trehalose. **(D,E)** Effect of sugar sources (200 mM mannitol, sucrose, cellobiose, or trehalose) on production of non-essential amino acids **(D)** and essential amino acids **(E)** in the presence of 10 mM glutamine. **(F,G)** Synergetic effects of sugar (200 mM trehalose) and nitrogen (10 mM glutamine) sources on production of non-essential amino acids **(F)** and essential amino acids **(G)**. Means ± s.e.m are shown (*N* = 4). Different letters (a, b) on the bars indicate statistically significant differences (*p* < 0.05, likelihood ratio test for GLM and posthoc multiple comparisons).

To evaluate quantitative aspects of EAA production in the midgut symbiotic system of *M. punctatissima*, we developed an *in vitro* culture system of the isolated symbiotic midgut. The main symbiotic midgut (Figure 1B), in which host-symbiont nutritional exchange mainly takes place (Koga et al., 2021), was ligated and dissected from adult *M. punctatissima*, and incubated in a hanging drop of culture medium (Figure 5A). The culture medium was modified from Buffer A, which was originally developed for culturing aphid bacteriocytes (Sasaki and Ishikawa., 1995).

First, we explored carbon and nitrogen sources suitable for efficient production of amino acids by the cultured symbiotic organ. Glutamate plays a key role in the synthesis of most amino acids as an amino group donor. However, the concentration of glutamate was low in the hemolymph, while its amide form, glutamine was abundant (Figure 3A). Thus, in the first culture trial, 1 mM glutamate or 10 mM glutamine was added to the medium. The supply of glutamine at the physiological concentration facilitated amino acid production encompassing both non-EAAs and EAAs, while the supply of glutamate was less effective (Figures 5B,C). These results suggest that the symbiotic midgut receives glutamine as the main nitrogen source, which is probably converted to glutamate after incorporation into the symbiotic organ.

Next, we tested the influence of various sugars as carbon source for amino acid production in the presence of 10 mM glutamine. We compared a hemolymphal disaccharide trehalose (see Supplementary Table S2), phloem-derived disaccharide sucrose, undigestible disaccharide cellobiose, and non-metabolizable sugar alcohol mannitol. As expected, trehalose most efficiently facilitated amino acid production in the symbiotic organ, whereas sucrose was certainly utilized for amino acid production but less efficiently (Figures 5D,E). These results favor the idea that the symbiotic midgut has an optimized metabolic mechanism to efficiently utilize the hemolymphal sugar source. In previous physiological studies on aphid endosymbiosis, sucrose was used for culturing symbionts or symbiotic organs (Sasaki and Ishikawa, 1995; Russel et al., 2013). However, our results suggest that the use of trehalose may provoke more natural metabolic pathways and improve production efficiency of amino acids and other metabolites.

Next, we examined the synergetic effect of glutamine and trehalose, and confirmed that the presence of both glutamine and trehalose maximized amino acid production (Figures 5F,G). Glutamine solely enabled the symbiotic organ to generate certain levels of amino acids, probably because it can serve as both nitrogen and carbon sources, while trehalose alone failed to induce amino acid synthesis effectively, likely due to the absence of amino group.

**FIGURE 6:**
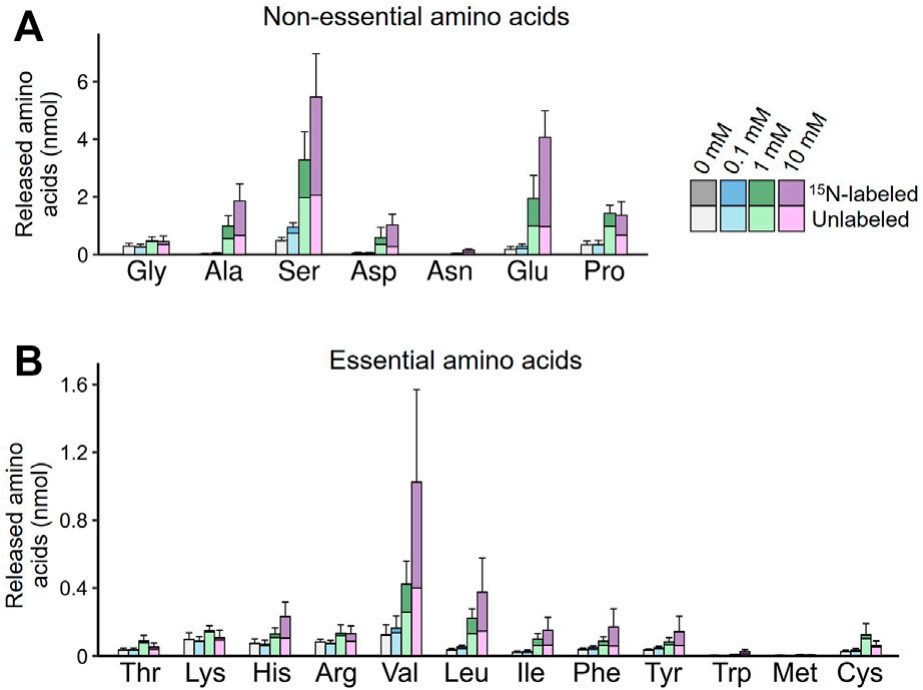
*De novo* production of amino acids in the isolated midgut symbiotic organ of *M. punctatissima*. The isolated midgut preparations were individually cultured with ^15^N-labeled glutamine at different concentrations (0–10 mM) in the presence of 200 mM trehalose. The graphs show mean ± s.e.m (*N* = 4) for non-essential amino acids **(A)** and essential amino acids **(B)**, with the labeled and unlabeled fractions coded by different colors.

We further obtained direct evidence of *de novo* EAA synthesis in the symbiotic organ by using [^15^N]-glutamine labeled at the amino group. The dissected symbiotic organs were incubated in the culture media supplemented with 0–10 mM [^15^N]-glutamine, and amino acids released into the culture media were analyzed by mass spectrometry. At least a part of newly synthesized amino acids was predicted to receive the labeled amino group in a transamination process from [^15^N]-glutamate derived from [^15^N]-glutamine. Figure 6 illustrates the labeled and unlabeled fractions for each of released non-EAAs and EAAs. As expected, the higher concentration of [^15^N]-glutamine was supplemented, the higher amount and proportion of labeled glutamate was released into the culture media. Similar patterns of [^15^N]-labeling were observed in the other released amino acids including EAAs. In the presence of 10 mM [^15^N]-glutamine, significantly higher amounts of [^15^N]-labeled amino acids were detected for all EAAs, except for methionine, when compared with the control group without glutamine supply (*p* < 0.05, a likelihood ratio test for GLM). The amounts of the released amino acids greatly varied from 7.6 nmol (cysteine) to 624.0 nmol (valine). Notably, branched-chain amino acids (valine, leucine, isoleucine), aromatic amino acids (phenylalanine, tyrosine), and histidine were preferentially released into the culture media. These results demonstrate that the symbiotic midgut possesses a *de novo* synthesizing ability of EAAs, where the specific EAAs deficient in plant sap diet are preferentially produced from hemolymph-derived nutritional sources.

It should be noted that this *in virto* culture system may not perfectly mimic the *in vivo* conditions. For example, our medium did not contain a sulfur source, which may be a reason for the low levels of methionine and cysteine production in our experiment (also see Douglas, 1988), in addition to the reason that these amino acids are intrinsically low-abundance components.

### Convergent role of midgut symbiotic organ for EAA production

All these results taken together, we conclude that the gut symbiont *Ishikawaella* plays a pivotal role for the host stinkbug by supplementing EAAs that are deficient in the phloem sap diet. Notably, while the *Ishikawaella* genome retains almost all genes required for synthesis of EAAs (except for a few final step transaminases like *ilvE*) (Nikoh et al., 2011), the EAAs produced by *Ishikawaella* are quantitatively not uniform but varied considerably, and interestingly, the production patterns of EAAs are skewed to meet the nutritional demand of the host stinkbug, presumably as a consequence of cooperative metabolic interactions in the midgut symbiotic organ. Similarly, biased production of symbiont-derived EAAs has been observed in the aphid-*Buchnera* symbiotic system (Sasaki and Ishikawa, 1995; Douglas et al., 2001; Akman Gündüz and Douglas, 2012). Evidently, the extracellular symbiosis in the midgut symbiotic organ of the plataspid stinkbug is structurally, physiologically and evolutionarily distinct from the intracellular symbiosis within the bacteriocytes of the aphid (Douglas, 2020). Hence, it seems quite likely that the functional similarities between them have evolved convergently through adaptation to the phloem-sap feeding lifestyle shared between them. The similarities and differences between the two symbiotic systems are of interest and discussed below.

### Regulation of amino acid production by host symbiotic organ

The results of *in vitro* culture experiments showed that EAA production in the symbiotic organ is efficiently fueled by glutamine and trehalose that are abundantly present in the hemolymph of *M. punctatissima*. In the aphid, the bacteriocytes are also known to uptake glutamine as a nitrogen source for EAA production via conversion to glutamate (Sasaki and Ishikawa, 1995) and sugars (probably trehalose converted from dietary sucrose) for a source of carbon skeleton (Febvey et al., 1999). Considering that the bacteriocyte in the aphid and the symbiotic midgut epithelium in the stinkbug are commonly lying between the insect body and the symbiont cells, it seems likely that they have common biological roles to adjust EAA production through nutritional allocation (MacDonald et al., 2011; Smith and Moran., 2020; Wilson, 2020). In the aphid system, a glutamine transporter preferentially expressed in the bacteriocytes was highlighted as a candidate regulator with a feedback control mechanism (Price et al., 2014). We observed that the amount of released EAAs from the symbiotic midgut is dependent on ambient glutamine concentration (Figure 6), which suggests the possibility that adjustment of EAA production may be feasible via controlling the uptake rate of glutamine in the stinkbug system. In this context, future studies should direct to identification of transporter genes responsible for glutamine uptake in the midgut symbiotic organ of the stinkbug. Besides glutamine, other hemolymphal amino acids may function as nitrogen carriers for EAA synthesis. Especially, proline is a promising candidate because it is present in the stinkbug hemolymph at high concentrations (Figure 3A) and can be readily converted to glutamate in the host cells (Bursell, 1977; Scaraffia and Wells, 2003).

### Host-symbiont nutritional exchange

It was reported that isolated *Buchnera* cells from the aphid bacteriocytes uptake glutamate rather than glutamine (Sasaki and Ishikawa, 1995). In contrast, a flux balance analysis predicted that *Buchnera* cells more actively uptake glutamine rather than glutamate (MacDonald et al., 2012). In *M. punctatissima*, glutamine taken up from the hemolymph is promptly converted to glutamate within the symbiotic organ (Figure 6A), and therefore both forms are potentially available for *Ishikawaella*. However, we presume that *Ishikawaella* has a higher requirement of glutamine for the following reasons: (i) *Ishikawaella* is incapable of synthesizing glutamine from glutamate, while glutamate can be produced from glutamine as a byproduct of nucleic acid synthesis (Nikoh et al., 2011); (ii) The amide group of glutamine is required for the synthesis of various nitrogenous metabolites like tryptophan and purines; and (iii) In *Ishikawaella*, as in *Buchnera*, the majority of glutamate-consuming transamination processes are likely to take place within the host cell on account of the lack of aminotransferases for EAA synthesis in the symbiont genome. Hence, it is plausible that *Ishikawaella* preferentially receives the amino source in the form of glutamine from the host cell. In addition, some non-EAAs (asparagine, serine, and proline) and some EAAs (transamination product of valine, leucine, and isoleucine) are predicted to be supplied by the host due to the absence of their synthetic capabilities (Nikoh et al., 2011).

Many, if not all, genome-reduced intracellular symbiotic bacteria like *Buchnera* lack the majority of genes constituting the TCA cycle (Shigenobu et al., 2000; McCutcheon and Moran, 2012). By contrast, *Ishikawaella* retains intact pathways for carbohydrate metabolism including the complete TCA cycle (Nikoh et al., 2011). Thus, the supply of sugar is enough for *Ishikawaella* to fuel energy production and carbon skeleton synthesis of EAAs. Due to the lack of trehalase genes, *Ishikawaella* may receive monosaccharides (presumable glucose cleaved from trehalose) from the host. Glutamate and other non-EAAs can be incorporated into the TCA cycle after deamination, which also contribute to the carbohydrate metabolism. Retainment of the TCA cycle genes in *Ishikawaella* may be a constraint associated with its extracellular habitat, where metabolic intermediates are not so readily available as in the host cytosol for intracellular symbionts (Nikoh et al., 2011). Meanwhile, some gut symbionts of other stinkbugs have lost a part of the TCA cycle genes (Kaiwa et al., 2014; Otero-Bravo and Sabree., 2018), suggesting that some of the metabolic intermediates may be potentially exchangeable in such extracellular symbiotic relationships. In any case, further studies are needed to clarify what metabolites are delivered to the symbiotic bacteria in the gut lumen of the stinkbug.

### Tyrosine is the most limited amino acid

**FIGURE 7:**
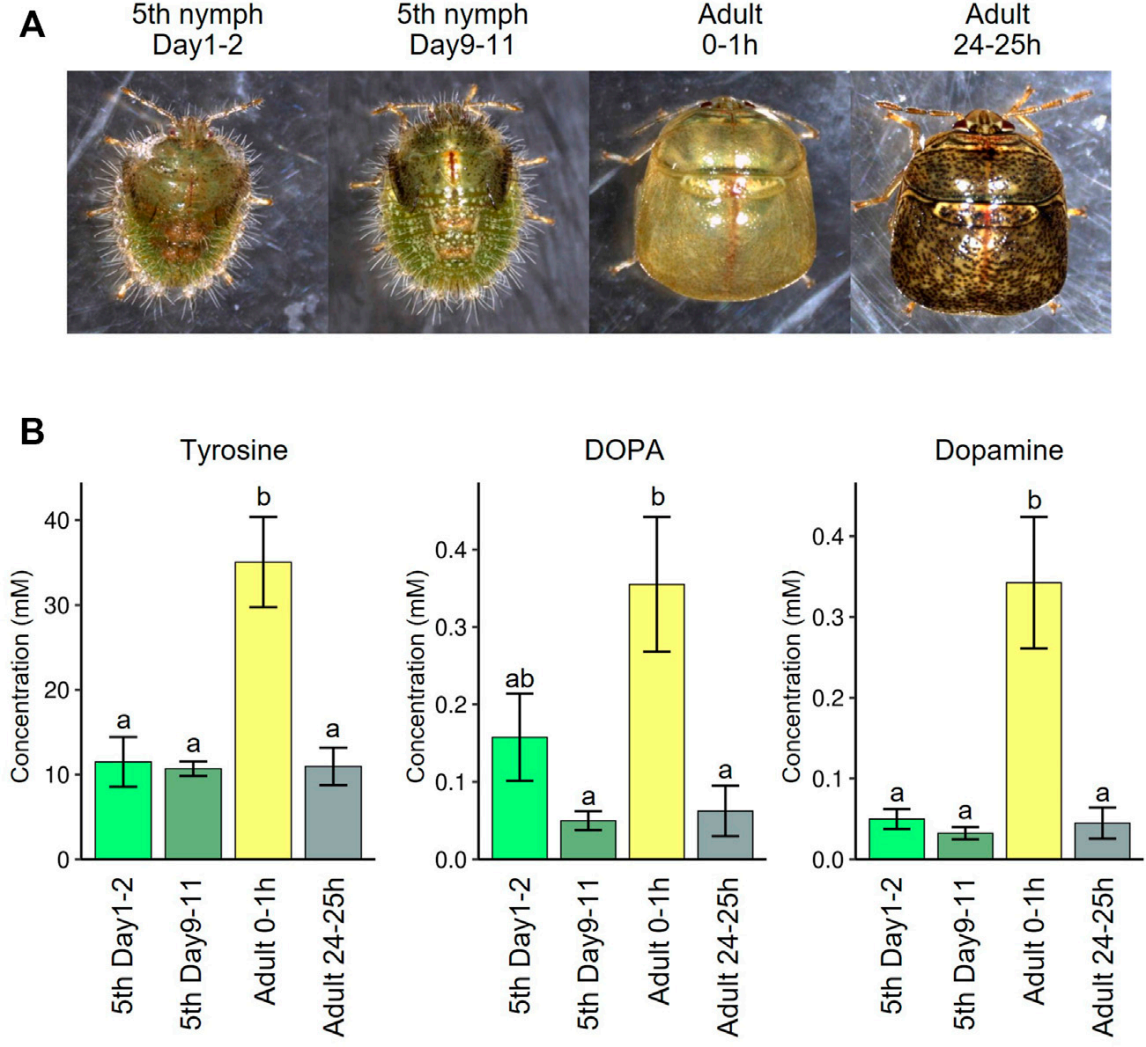
Tyrosine recruitment during cuticle pigmentation upon adult emergence of *M. punctatissima*. **(A)** Photos of fifth instar nymphs and adults before and after adult molting. **(B)** Hemolymph titers of tyrosine and its derivatives, DOPA and dopamine, before and after adult molting. To liberate from their conjugated forms (Hopkins et al., 1998), the hemolymph samples were treated with 6 N hydrogen chloride solution at 105°C for 24 h. The graphs show mean ± s.e.m. (*N* = 4). Different letters (a,b) indicate statistically significant differences (*p* < 0.05, likelihood ratio test for GLM and posthoc multiple comparisons).

Which amino acid is the most limiting for *M. punctatissima* development? In aphids, tryptophan is often deemed the most pivotal shortfall in the phloem sap diet (Bernays and Klein, 2002; Bermingham and Wilkinson, 2010), while disruption of phenylalanine supply exerts more detrimental effects on aphid survival (Douglas et al., 2001; Simonet et al., 2016), although both nutritional demands and dietary amino acid compositions are variable with physiological and genetic conditions of both insect and plant sides (Douglas, 1996; Sandström and Moran, 2001). In *M. punctatissima*, tyrosine was the only protein amino acid that was significantly depressed in *Ishikawaella*-free insects (Figure 4B), and consistently decreased in the free amino acid constituents of the whole body and excreta along with its precursor phenylalanine (Figures 4D,F). However, in the view of nutritional adequacy calculated based on phloem sap compositions against body protein compositions, tyrosine/phenylalanine were not the sole shortfalls among EAAs (Figure 2B). This discrepancy likely comes from its extra need for something other than protein construction. In insects, derivatives of tyrosine are exploited for neural activity, immune reaction, and cuticle pigmentation/sclerotization (Vavricka et al., 2014). Tyrosine/phenylalanine synthetic pathway (= shikimate pathway) is widely conserved among many insect symbiont genomes. In these insects, symbiont-produced tyrosine/phenylalanine is shown to be indispensable for insect growth and survival (Douglas et al., 2001; Rabatel et al., 2013; Vigneron et al., 2014; Simonet et al., 2016; Kutsukake et al., 2019). As extreme cases, some symbiont genomes of beetles and ants are so highly streamlined that, besides the minimal gene set needed for basic cellular activities, the tiny genomes virtually retain the tyrosine/phenylalanine synthesis pathway only (Klein et al., 2016; Anbutsu et al., 2017; Kiefer et al., 2021). It was experimentally proven that the symbiont-provisioned tyrosine is responsible for pigmentation and sclerotization of their hard cuticle (Anbutsu et al., 2017; Kiefer et al., 2021). Also in *M. punctatissima*, previous studies reported that symbiont-deprived insects exhibit abnormal behavior, retarded growth, low adult emergence, smaller body size, infertility, and paler body color with reduced melanization (Fukatsu and Hosokawa, 2002; Hosokawa et al., 2006, 2008). It is also notable that RNAi targeting a multicopper oxidase (= laccase2), which catalyzes tyrosine-based cuticle sclerotization and pigmentation, causes failure of cuticle pigmentation and ecdysis in *M. punctatissima* and other stinkbugs (Futahashi et al., 2011; Nishide et al., 2020). In fact, we confirmed that the levels of tyrosine and its derivatives (DOPA and dopamine) in the hemolymph of *M. punctatissima* are remarkably elevated during the period of cuticle pigmentation and sclerotization just after adult ecdysis (Figure 7) as observed in other insects (Hopkins, 1982). These results and observations strongly suggest that symbiont-supplemented tyrosine is pivotal for normal morphogenesis and pigmentation of *M. punctatissima*. The high demand for tyrosine may reflect the necessity for constructing the hard and pigmented exoskeleton of the stinkbug (see Figure 1A), which is in contrast to the soft and transparent cuticle of the aphid. In this context, it should be noted that *Ishikawaella* possesses both tyrosine and phenylalanine pathway genes (Nikoh et al., 2011) whereas *Buchnera* has lost the tyrosine branch (Shigenobu et al., 2000). This is also consistent with the higher tyrosine demand in the stinkbug, but it remains unknown whether this difference affects the total production of tyrosine and phenylalanine.

### Conclusion and perspective

The present study figured out the cooperative host-symbiont metabolic interactions that compensate for the nutritional imbalance in the phloem sap-feeding stinkbug with an extracellular gut symbiotic system. We uncovered a convergent functional evolution of the mutualistic relationship across the different insect groups with phloem sap-feeding lifestyle, the stinkbug and the aphid, in spite of their distinct symbiotic systems entailing the extracellular association within gut cavity vs. the intracellular association within the cytoplasm of host cells. The stinkbug-*Ishikawaella* gut extracellular symbiosis will provide a new experimental model system with which some of the limitations of the conventional model system, the aphid-*Buchnera* intracellular symbiosis, can be overcome in the following respects: (i) The extracellular symbiotic bacteria are localized to a compartmentalized space outside of the host cells. Hence, experimental handling, separation and manipulation of the host-symbiont relationship are easier to carry out. The host-symbiont interface can be defined and observed more clearly in the gut extracellular association, which is suitable for experimental analysis and visualization of host-symbiont molecular interplay. (ii) The extracellular symbiotic bacteria are excreted and placed besides the eggs upon oviposition for vertical transmission to the offspring (Salem et al., 2015; Hosokawa and Fukatsu, 2020), which enables convenient disruption, replacement and manipulation of the host-symbiont association (Hosokawa et al., 2007; Hosokawa et al., 2016), although successful experimental symbiont replacement has been reported in the aphid-*Buchnera* system (Moran and Yun, 2015). (iii) Some of the extracellular symbiotic bacteria of stinkbugs are cultivable and genetically manipulatable (Kikuchi et al., 2007; Kikuchi and Fukatsu, 2014; Hosokawa et al., 2016; Koga et al., 2022), which enables molecular genetic approaches to the symbiont functioning. (iv) Among diverse stinkbugs in general, RNA interference by double-stranded RNA injection efficiently knockdown the target gene expression (Futahashi et al., 2011; Koga et al., 2021; Moriyama et al., 2022), which enables molecular genetic approaches to the mechanisms of symbiosis also from the host side. The stinkbug-bacterium gut symbiotic system will provide a promising tool to deepen the current understanding of physiology, mechanism and functioning of intimate nutritional mutualism, and this study has established a basic framework for it.

## Data Availability

The original contributions presented in the study are included in the article/Supplementary Material further inquiries can be directed to the corresponding authors.
